# Propolis in Metabolic Syndrome and Its Associated Chronic Diseases: A Narrative Review

**DOI:** 10.3390/antiox10030348

**Published:** 2021-02-26

**Authors:** Felix Zulhendri, Munir Ravalia, Krishna Kripal, Kavita Chandrasekaran, James Fearnley, Conrad O. Perera

**Affiliations:** 1 Kebun Efi, North Sumatra 2217, Indonesia; 2 The Royal London Hospital, Whitechapel Rd, Whitechapel, London E1 1FR, UK; 3 Rajarajeswari Dental College & Hospital, No.14, Ramohalli Cross, Mysore Road, Kumbalgodu, Bengaluru 560074, Karnataka, India; kripalkrishna@yahoo.com; 4 Peerzadiguda, Uppal, Hyderabad 500039, Telangana, India; dr.ckavita@gmail.com; 5 Apiceutical Research Centre, Unit 3b Enterprise Way, Whitby, North Yorkshire YO18 7NA, UK; james.fearnley@beearc.com; 6 Food Science Program, School of Chemical Sciences, University of Auckland, 23 Symonds Street, Auckland CBD, Auckland 1010, New Zealand

**Keywords:** propolis, metabolic syndrome, antioxidant, anti-inflammation, chronic diseases, cardiovascular, diabetes mellitus, chronic kidney disease, fatty liver disease, Alzheimer’s disease

## Abstract

Propolis is a resinous product collected by bees from plants to protect and maintain the homeostasis of their hives. Propolis has been used therapeutically by humans for centuries. This review article attempts to analyze the potential use of propolis in metabolic syndrome (MetS) and its associated chronic diseases. MetS and its chronic diseases were shown to be involved in at least seven out of the top 10 causes of death in 2019. Patients with MetS are also at a heightened risk of severe morbidity and mortality in the present COVID-19 pandemic. Propolis with its antioxidant and anti-inflammatory properties is potentially useful in ameliorating the symptoms of MetS and its associated chronic diseases. The aim of this article is to provide a comprehensive review on propolis and its therapeutic benefit in MetS and its chronic diseases, with an emphasis on in vitro and in vivo studies, as well as human clinical trials. Moreover, the molecular and biochemical mechanisms of action of propolis are also discussed. Propolis inhibits the development and manifestation of MetS and its chronic diseases by inhibiting of the expression and interaction of advanced glycation end products (AGEs) and their receptors (RAGEs), inhibiting pro-inflammatory signaling cascades, and promoting the cellular antioxidant systems.

## 1. Introduction

The top ten causes of death in 2019 listed by the World Health Organization (WHO) showed seven out of 10 leading causes of death were non-communicable diseases, namely, ischemic heart disease, stroke, Alzheimer’s disease (and other dementias), cancers, diabetes mellitus, and kidney disease [1]. Metabolic syndrome (MetS) has been shown to significantly increase the risk of these chronic diseases [2,3,4,5,6,7,8,9,10]. The economic burden to the health service of a patient with MetS is often a few-fold higher compared to a patient without MetS [11]. MetS and its associated chronic diseases also greatly increase morbidity and mortality in viral infections, such as COVID-19 [12,13,14,15]. Consequently, MetS has become a major public health challenge all around the world [16,17,18].

MetS is a cluster of risk factors; abdominal obesity (population specific), increased triglycerides (≥150 mg/dL or ≥1.7 mmol/L), reduced HDL (<40 mg/dL for men and <50 mg/dL for women), increased blood pressure (systolic ≥ 130 mm Hg and/or diastolic ≥ 85 mm Hg), and increased fasting glucose (>100 mg/dL or 5.5 mmol/L) [19,20,21]. Lifestyle and dietary factors are considered the main drivers of MetS [22,23,24]. In addition to lifestyle changes, nutraceutical supplementations such as micronutrients, pro- and prebiotics, polyphenols, plant extracts, and other natural products have been shown to be effective in ameliorating the severity of MetS symptoms [25,26,27,28,29,30,31,32].

The present study attempts to review the potential therapeutic use of propolis in MetS and its associated chronic diseases. Propolis is plant resin collected by bees. The precise composition of propolis varies depending on the source. It comprises wax, resin, balsam, essential oils, pollen, and other bioactive compounds such as amino acids, minerals, vitamins, phenolics, flavonoids, and terpenoids [33,34,35]. The exact content of propolis is variable depending on the source [36]. Propolis is important in the survival of a bee colony as it serves several crucial functions, namely, maintenance of hive homeostasis, physical protection, antimicrobial action, detoxification processes, immune modulation, and stabilization of the beneficial microbiome [37,38,39,40,41,42].

The oldest record of the use of bee products by humans dates back to c. 13,000 BCE. Ancient Egyptians, Greeks and Romans used propolis to alleviate many ailments [43]. Recent studies have demonstrated that propolis has a wide range of therapeutic and health benefits for humans, such as antibacterial, antiviral, anti-inflammation, antioxidant, and antiproliferative effects [44,45,46,47,48,49]. Recently, propolis has also been shown to be a potential therapeutic agent against SARS-CoV-2 and COVID-19 disease [50,51,52]. The term “propolis” in this review study includes propolis from all propolis-producing bees, namely, European honey bees (*Apis mellifera*), Asian honey bees (*Apis cerana*), and stingless bees of the genera *Trigona*, *Melipona, Geniotrigona*, *Heterotrigona*, and *Tetragonula*.

## 2. Cardiovascular Diseases (CVDs)

### 2.1. Atherosclerosis

Atherosclerosis is the major cause of CVD and has been shown to be strongly associated with oxidation and inflammation. Increased production of reactive oxygen species (ROS) damages the functions of cellular lipids, proteins, and carbohydrates. Increased ROS also induces lipid peroxidation. Oxidation of lipoproteins is the initial stage of atherosclerosis. The development and progression of the pathophysiology of atherosclerosis have been shown to significantly involve the dysregulation of immune-related physiology and inflammatory responses [53,54,55,56,57]. There is an ongoing debate with regard to which lipoprotein is the most important in the formation of atherosclerotic plaque: low-density lipoproteins (LDLs), small dense LDL, VLDL, or remnant cholesterol [58,59,60].

Oxidized lipoproteins promote endothelial cell damage and endothelial dysfunction. The endothelial dysfunction causes the recruitment of leukocytes in subendothelial space of the tunica intima (the innermost layer of an artery) and induces the secretion of pro-inflammatory cytokines such as tumor necrosis factor alpha (TNF-*α*), interleukin-1 (IL-1), IL-4, IL-6, and interferon gamma (IFN-*γ*). The cytokines upregulate the expression of various leukocyte adhesion molecules such as vascular cell adhesion molecule-1 (VCAM-1), intercellular adhesion molecule-1 (ICAM-1), and E-selectin [61]. The adhesion molecules further recruit and bind to circulating monocytes and T-lymphocytes. Monocytes, in the subendothelial space, subsequently differentiate into macrophages through monocyte chemotactic protein-1 (MCP-1), macrophage colony-stimulating factor (M-CSF), and IL-8. These macrophages recognize and scavenge the oxidized lipids which results in the formation of foam cells. These foam cells then undergo apoptosis and create cellular debris and lipids [53,54,55,56,57]. The resulting inflammatory cascades and lipid-laden foam cells release various cytokines and growth factors that promote the migration of smooth muscle cells (SMCs) from middle layer of the artery walls (tunica media) to the tunica intima. Once in the intima, the SMCs then proliferate and produce an array of extracellular matrix (ECM) in the process of atheroma formation which consequently blocks blood vessels. SMC-derived ECM forms a fibrous cap over the plaque. The collagen degradation of the ECM would consequently rupture the plaque, which triggers the release of pro-coagulation factors that lead to thrombosis and myocardial infarction [53,54,55,56,57].

Propolis has been shown to have cardioprotective properties. Claus et al. (2000) showed that the aqueous extract of propolis and its component, propol, reduced Cu^2+^-induced oxidation of LDL in vitro. They also showed that the extract reduced oxidized LDL-induced macrophage apoptosis [62]. Propolis extract reduced the activity of activated macrophages and the expression of the matrix metalloproteinase-9 (MMP-9) gene in a dose-dependent manner [63]. Matrix metalloproteinases are involved in the degradation of proteins and proteoglycan components of extracellular matrix (ECM) which contributes to the pathogenesis of atherosclerosis [64]. Propolis and its polyphenols were also shown to inhibit platelet aggregation (anticoagulation) in vitro [65].

The cardioprotective property of propolis was demonstrated in various animal studies. Daleprane et al. (2012) found that three types of Brazilian propolis extracts, red, green, and brown, increased the expression of the metalloproteinase inhibitor TIMP-1 while reducing the expression of VCAM, MCP-1, fibroblast growth factor (FGF), platelet-derived growth factor (PDGF), vascular endothelial growth factor (VEGF), platelet endothelial cell adhesion molecule (PECAM), and MMP-9 genes in LDL receptor gene (LDLr-/-) knockout mice [66]. They demonstrated that all three types of propolis significantly inhibited the early development atherosclerotic lesions. However, only red propolis inhibited the advanced atherosclerotic lesions [66], highlighting the need for characterization and standardization of the active compounds of propolis.

MicroRNAs (miRNAs) involved in the development or attenuation of atherosclerotic plaques in LDLr-/- mice were shown to be modulated by propolis [67]. Propolis upregulated the expression of miR-181a, miR-106a, and miR-20b, which correlated with the inhibition of VEGFA and hypoxia-inducible factor-1 (Hif1a) expression [67]. VEGFA is one of the main pro-angiogenic factors in atherosclerotic lesions and Hif1a is expressed in the necrotic nucleus of the atheroma [67,68,69]. Propolis prevented left ventricular hypertrophy (LVH), the formation of atherosclerotic lesions, arterial and ventricular inflammation, and CD40L expression in LDLr-/- mice [70]. Propolis also increased the serum HDL-c level in the LDLr-/- mice [70]. Fang et al. (2013) demonstrated that ethanolic extract of propolis reduced total cholesterol, triglycerides, and non-HDL-C by 21%–32% compared to control in ApoE (ApoE-/-) knockout mice [71]. Propolis also reduced the expression of pro-inflammatory cytokines, IL-6 and IL-17, and vasoconstrictor peptide endothelin. More importantly, propolis reduced the formation of atherosclerotic lesions in the aortic root and whole aorta [71].

The efficacy of propolis in preventing cardiovascular diseases has also been demonstrated in various human clinical trials. Mujica et al. (2017) carried out a randomized placebo-controlled trial and found that propolis supplementation for 90 days increased HDL-c from 53.9 ± 11.9 to 65.8 ± 16.7 mg/dL. It also increased serum glutathione (GSH) by 175% and reduced the thiobarbituric acid reactive substance (TBARS) level by 67% [72]. The serum TBARS level has been shown to be an oxidative stress marker and a strong predictor for cardiovascular events [73]. In addition, Samadi et al. (2017) found that propolis prevented an increase in total cholesterol and LDL-c in type 2 diabetes patients [74]. However, Zakerkish et al. (2019) found that propolis did not reduce LDL-c and total cholesterol but instead increased the serum level of HDL-c in type 2 diabetes patients [75]. Furthermore, Hesami et al. demonstrated that propolis reduced the level of oxidized LDL in type 2 diabetic patients [76]. It appears propolis has a net positive effect in terms of increasing antioxidant status and improving the lipid profile.

### 2.2. Hypertension

Hypertension has been demonstrated to be related to many CVD outcomes including ischemic heart and cerebrovascular diseases. Hypertension was shown to account for 41% of all disability-adjusted life years (DALYs) in the Global Burden of Disease, Injuries, and Risk Factor Study 2015 (GBD 2015) [77]. More importantly, significant numbers of sufferers are not even aware of the condition. Furthermore, those diagnosed with hypertension are frequently given inadequate treatments. Consequently, hypertension will remain as one of major significant public health challenges worldwide regardless of income level [78,79,80].

Propolis and its phenolic and flavonoid components appear to have beneficial antihypertensive effects. Mishima et al. (2005) demonstrated that various extracts (water and ethanol) of propolis had hypotensive activity in spontaneously hypertensive rats [81]. It was shown that 25% ethanol extract was more efficacious than 70% ethanol. They also identified that di- and tri-caffeoylquinic acids were characteristic compounds responsible for the hypotensive effect in the 25% ethanol extract [81]. In addition, Maruyama et al. (2009) showed that ethanol-eluted fractions of propolis were effective in treating spontaneously hypertensive rats [82]. They isolated and identified four flavonoids, dihydrokaempferide, isosakuranetin, betuletol, and kaempferide, as being the most effective in treating hypertension in the spontaneously hypertensive rats. These flavonoids worked in a dose-dependent manner [82]. Furthermore, propolis was shown to exert an antihypertensive effect in Otsuka Long-Evans Tokushima fatty (OLETF) rats, without affecting the level of aldosterone, suggesting that the effect of propolis is not through endocrine signaling pathways [83].

Several mechanisms of action of the antihypertensive effect of propolis have been suggested. Gogebakan et al. (2012) investigated the antihypertensive effect of propolis (30% ethanol extract) in chronic nitric oxide synthase (NOS)-inhibited rats by *N*_w_-nitro-l-arginine methyl ester (L-NAME) [84]. Propolis extract was shown to reduce the tyrosine hydroxylase activity in the hypertensive L-NAME-treated rats. Tyrosine hydroxylase is a rate-limiting enzyme in the synthesis of catecholamine. Excessive secretion of catecholamine has been shown to promote the over-activation of the sympathetic nervous system, which contributes to hypertension [85]. Propolis also reduced oxidative stress in the hypertensive L-NAME-treated rats by decreasing the expression of malondialdehyde (MDA) [86]. Salmas et al. (2017) investigated the effect of propolis, caffeic acid phenethyl ester (CAPE), a propolis-derived compound, and bee pollen in hypertensive L-NAME-treated rats. All treated rats were shown to have better biochemical markers associated with oxidation and inflammation, namely, paraoxonase (PON1), oxidative stress index (OSI), total antioxidant status (TAS), total oxidant status (TOS), asymmetric dimethylarginine (ADMA), and nuclear factor kappa B (NF-κB) [87].

High-NaCl diet-induced hypertension has also been shown to be ameliorated by propolis. Zhou et al. (2020) demonstrated that water-soluble propolis extract reduced the severity of hypertension induced by a high-NaCl diet in rats [88]. They demonstrated that the protective effect of a water-soluble extract of propolis was through several modes of action, such as the upregulation of antioxidant enzyme catalase (CAT) activity and the reduction in the vascular ROS level. Propolis also reduced inflammatory markers, such as TNF-*α* and IL-6, and improved endothelial function [88]. Furthermore, Mulyati et al. (2021) investigated the antihypertensive effect of propolis collected from three different regions in Indonesia, namely, Riau Archipelago, Lampung, and South Sulawesi in NaCl-induced hypertensive rats. It was found that all three propolis extracts ameliorated hypertension. However, the antihypertensive effect was not uniform, with propolis from Riau Archipelago and South Sulawesi being more effective compared to propolis from Lampung [89]. This study illustrates the common problem in propolis research where chemical analysis data are often lacking. Consequently, it is difficult to pinpoint the specific chemical compounds or groups of chemical compounds responsible for the biological activity of propolis from different sources and/or geographical locations.

## 3. Type 2 Diabetes Mellitus

Non-insulin-dependent/type 2 diabetes mellitus is characterized by insulin resistance and/or abnormal insulin secretion (hyperinsulinemia), which subsequently cause abnormally high blood glucose levels (hyperglycemia) [90,91]. Chronic hyperglycemia damages and causes the failure of different organs, especially the eyes, kidneys, nerves, heart, and blood vessels [92,93]. MetS is a strong predictor for the development of type 2 diabetes [94,95,96].

Propolis ameliorated the symptoms of diabetes in animal models. Water-soluble and ethanolic extracts of propolis were shown to be effective in reducing the increase in blood glucose in alloxan-induced diabetic rats by 18.2%–29.5% in an 8-week experiment [97]. Ethanolic extract of propolis improved blood glucose control and insulin sensitivity in streptozotocin-induced diabetic rats [98,99]. Propolis also had a protective effect on pancreatic β-cells [100,101]. Propolis significantly reduced blood glucose and plasma insulin in Otsuka Long-Evans Tokushima fatty (OLETF) rats (spontaneously diabetic rats) [83]. Propolis extract reduced blood glucose and improved glucose tolerance and insulin sensitivity in *ob/ob* mice independent of changes in body weight and food intake [102]. It appeared that the blood glucose reduction by propolis was dose-dependent [103].

Furthermore, the efficacy of propolis in reducing pre-diabetic and diabetic symptoms was evident in human clinical trials. Fukuda et al. (2015) investigated the effect of supplementing type 2 diabetes patients with 226.8 mg Brazilian green propolis daily for 8 weeks [104]. They found that the propolis supplement prevented diabetic patients from developing worse blood uric acid and estimated glomerular filtration rate (eGFR). Zhao et al. (2016) demonstrated that propolis improved antioxidant status in type 2 diabetes patients [105]. Serum GSH in the propolis group was increased by ~174% at the end of an 18-week trial period. A reduction in other oxidative and inflammatory parameters, such as serum carbonyls (~17% reduction), TNF-α (~21% reduction), and lactate dehydrogenase activity (~8% reduction), was also observed. However, they noted an increase in IL-6 and IL-1β. They also did not find an improvement in glucose metabolism [105]. A propolis-induced increase in antioxidant parameters in type 2 diabetes patients was confirmed by Gao et al. (2018) [106]. They found that after 18 weeks of propolis supplementation, serum GSH was increased by ~236% in the propolis group when compared to the placebo group [106].

El-Sharkawy et al. (2016) found that 400 mg daily propolis supplementation for 6 months significantly improved clinical parameters of type 2 diabetes patients. HbA1c was reduced by ~11.0% after 6 months. A similar trend was observed in fasting blood glucose. In addition, serum N^€^-(carboxymethyl) lysine (CML) was reduced by ~17% after 6 months [107]. They also found significant improvement in periodontal status in the propolis group patients [107]. Samadi et al. (2017) found that 900 mg/day of propolis consumption for 12 weeks reduced fasting blood glucose (by ~12%) and HbA1c (by ~9%) in type 2 diabetes patients [74]. Furthermore, Zakerkish et al. (2019) investigated the therapeutic benefit of supplementing type 2 diabetes patients with 1000 mg propolis/day for 12 weeks [75]. Propolis significantly improved glucose metabolism and reduced clinical parameters. Propolis reduced serum levels of HbA1c by ~11%, insulin by ~46%, homeostasis model assessment of insulin resistance (HOMA-IR) by ~39%, homeostasis model assessment of β-cell function (HOMA-β) by 42%, and serum TNF-α by ~30%. Propolis also prevented an increase in 2 h postprandial blood glucose and high-sensitivity C-reactive protein (hs-CRP) level throughout the trial period [75].

## 4. Chronic Kidney Disease

Studies have shown a strong association of MetS with the development and severity of renal dysfunction and chronic kidney disease (CKD) [108,109,110]. MetS patients have a several-fold higher risk of developing CKD and it is often shown that renal dysfunction manifests earlier than diabetes in MetS patients [111]. Patients with MetS often suffer from hyperhomocysteinemia, hyperuricemia, lower glomerular filtration rate (GFR), and increased albumin excretion [112,113].

Propolis has been shown to have renal protective properties in animal models. Propolis significantly reduced malondialdehyde (MDA) and increased antioxidant parameters, such as glutathione (GSH), superoxide dismutase (SOD) and catalase (CAT) activities, in renal tissue of streptozotocin-induced diabetic rats [103,114]. Propolis also maintained the antioxidant parameters of carbon tetrachloride (CCl_4_)-, diatrizoate-, and *N*_w_-nitro-l-arginine methyl ester (L-NAME)-induced oxidative-stressed in murine renal tissues [115,116,117,118]. The antioxidant properties were dose-dependent and correlated to the polyphenol contents of the propolis extract [103,115].

Propolis has a protective effect on the membrane integrity of renal tissue. Bhadauria (2012) showed that CCl_4_-damaged murine renal tissue treated with propolis retained better kidney histoarchitecture, less glomerulus swelling, and more uniform space between the glomerulus and capsule wall, when compared to CCl_4_-damaged murine renal tissue without propolis treatment [116]. The activity of renal membrane-bound enzymes such as adenosine triphosphatase (ATPase) and alkaline phosphatases (ACPase and ALPase) were considerably restored [116]. Propolis maintained the thickness of the glomerular basement membrane in renal tissues of diabetic rats [114]. Untreated diabetic rats had a significant increase in glomerular basement membrane thickness [114]. Propolis also reduced the CCl_4_-induced apoptosis of renal cells by downregulating the caspase-9 gene and upregulating Bcl-2 gene expression [119].

Furthermore, propolis attenuated methotrexate-induced renal injury [120]. Propolis treatment following methotrexate reduced the rate of apoptosis of renal cells and the degradation of renal morphology, compared to untreated controls [120]. Propolis also had a protective effect on gentamicin-induced renal injury. Propolis significantly reduced gentamicin-induced blood urea nitrogen levels, tubular injury, collagen and reticular deposition, and apoptosis of renal cells [121].

Propolis ameliorated proteinuria, serum creatinine retention, glomerulosclerosis, renal macrophage infiltration, and oxidative stress in 5/6 renal ablated rats (Nx) [122]. Propolis also appears to have a protective role in acute kidney injury. Propolis protected the kidney against ischemic–reperfusion acute renal injury by reducing oxidative stress and upregulating endothelial nitric oxide synthase and heme-oxygenase [123]. Histological analysis showed that propolis-treated renal tissue after ischemic–reperfusion had a significantly lower tubular necrosis score [123]. Propolis also inhibited pro-inflammatory signaling pathways, namely, SMAD 2/3-dependent and SMAD-independent JNK/ERK activation in the signaling cascades of TGF-β family, that have been implicated in the development of tubulointerstitial fibrosis in advanced chronic kidney disease in animal models [124].

The effect of propolis in ameliorating kidney disease was evident in human clinical trials. Silveira et al. (2019) demonstrated that the consumption of 500 mg/day of standardized propolis extract significantly reduced proteinuria and the urinary level of monocyte chemoattractant protein-1 (MCP-1), an inflammation marker, in a randomized, double-blind, placebo-controlled trial in patients with chronic kidney disease [125]. It was found that at the end of the trial period (12 months), proteinuria was significantly lower in the propolis arm, 695 mg/24 h, compared to 1403 mg/24 h in the placebo arm. Urinary MCP-1 was also reduced in the propolis arm (58 pg/mg creatinine) compared to the placebo arm (98 pg/mg creatinine) [125]. The same group also showed that propolis was effective in reducing high-sensitivity C-reactive protein (hs-CRP) in hemodialysis patients [126]. Both studies demonstrated that propolis was safe for patients with kidney disease and no adverse events were recorded [125,126].

## 5. Non-Alcoholic Fatty Liver Disease (NAFLD)

NAFLD is the major chronic liver disease in the world and represents the hepatic symptom of MetS [127,128]. NAFLD affects one in four adults and is expected to increase annually in parallel with the increase in MetS prevalence [129]. NAFLD is defined as the appearance of hepatic steatosis with the absence of causes for secondary hepatic fat accumulation, such as excessive alcohol consumption, use of steatogenic prescriptions, or any hereditary disorder [130]. NAFLD is then further categorized into two types; non-alcoholic fatty liver (NAFL) and non-alcoholic steatohepatitis (NASH) [130]. NAFL is diagnosed by the presence of hepatic steatosis without the ballooning of the hepatocytes, whereas NASH is defined by the evidence of hepatic steatosis and inflammation (hepatocyte ballooning) with or without the presence of fibrosis [130].

The hepatoprotective properties of propolis appear to be through antioxidant and anti-inflammatory pathways. Propolis and its polyphenols were shown to protect cultured hepatocytes from palmitic acid-induced lipotoxicity [131]. Propolis prevented palmitic acid toxicity of HepG2 cells by maintaining energy provision and inhibiting apoptosis. It also increased the antioxidant capacity of cells by upregulating the superoxide dismutase level and antioxidant gene expressions, such as GSTA1, TXNRD1, NQO-1, HO-1, and Nrf2, while reducing the expression of inflammatory genes of TNF-α and IL-8 [131]. Propolis-derived flavonoids, pinocembrin, galangin, and chrysin, prevented HepG2 cell injury by inhibiting ERK1/2-AHR-CYP1A1 signaling pathways [132]. ERK signaling pathways have been shown to be significant in the formation of liver fibrosis [133].

Propolis attenuated liver damage in an experimental model of diabetes and NAFLD in mice. Propolis reduced the level of alkaline phosphatase (ALP), alanine aminotransferase (ALT), aspartate aminotransferase (AST), lactate dehydrogenase (LDH), gamma-glutamyl transferase (GGT), and malondialdehyde in diabetic mice [134,135,136]^.^ Elevated liver enzymes have been shown to be strongly associated with the severity of liver disease [137]. Conversely, propolis increased the protective antioxidant status, upregulated antioxidant enzymes, namely, superoxide dismutase (SOD), catalase (CAT), glutathione peroxidase (GPx), glutathione-S-transferase (GST), and glutathione reductase (GR), and increased the glutathione level and hepatic total antioxidant capacity of diabetic mice [134,135,136]. Histological assessment also showed that propolis attenuated liver damage caused by diabetes. Propolis-treated diabetic mice had fewer vacuolized cells, a lesser degree of vacuolization, and less inflammation and infiltration of immune cells [134,135,136].

A similar trend was shown in NAFLD mice; propolis (and its component chrysin) ameliorated the symptoms by reducing AST, ALT, ALP, GGT, liver triglycerides, liver cholesterol, liver free fatty acids, and liver advanced glycation end products [138,139]. Propolis also lowers pro-inflammatory cytokines, TNF-α and IL-6, in NAFLD liver tissue [138,139]. Chrysin was shown to inhibit the expression of the SREBP-1c gene and upregulate the PPAR-α gene [139]. SREBP-1c plays an important role in the development of NAFLD, whereas PPAR-α directly inhibits the pro-inflammatory signaling pathways [140,141].

Even though the majority of the animal studies exhibited a hepatoprotective effect of propolis, Samie-Rad et al. (2014) demonstrated liver toxicity when very high doses (2000–8000 mg/kg) of propolis were given to mice [142]. The histopathological changes were dose-dependent and included central venous dilatation, steatosis, confluent necrosis, apoptosis, and focal necroinflammation [142]. However, the dose in this study was in the range of 10–40-fold that of the doses in the positive studies previously mentioned.

The hepatoprotective effect of propolis has been demonstrated in human clinical trials. Soleimani et al. (2020) demonstrated that propolis supplementation of 250 mg twice daily for 4 months had a beneficial effect among NAFLD patients [143]. A significant improvement of hepatic steatosis was evident in the treatment group compared to the placebo group. Propolis attenuated liver stiffness in the treatment group, whereas liver stiffness increased in the placebo arm. Propolis also reduced high-sensitivity C-reactive protein (hs-CRP) [143].

## 6. Alzheimer’s Disease (AD)

Alzheimer’s disease (AD) is characterized by the presence of amyloid plaques and intracellular neurofibrillary tangles (phosphorylated tau proteins). AD is the most common form of dementia and MetS has been shown to be highly correlated with the development of AD [144,145]. The elevated level of advanced glycation end products (AGEs) associated with hyperglycemia and hyperinsulinemia in MetS has been shown to promote the aggregation of β-amyloid (Aβ) and glycation and phosphorylation of tau proteins [146,147,148]. An increased level of oxidative stress in MetS damages mitochondria and promotes the development of AD [149,150]. An increased expression of pro-inflammatory cytokines (associated with MetS), such as TNF-α and IL-6, plays a significant role in the neuroinflammation and neurodegeneration associated with AD [9,151,152].

Antioxidant and anti-inflammatory properties of propolis play a major role in its neuroprotective capacity. Propolis protected microglia from hypoxia-induced inflammation and cytotoxicity by inhibiting nuclear factor kappa B (NF-κB) activation through the reduction of the expression of several key pro-inflammatory cytokines; IL-1β, IL-6, and TNF-α [153,154]. Caffeic acid phenethyl ester (CAPE), a propolis-derived compound, protected microglia by reducing the expression of neurotoxic factors iNOS and COX-2, and inflammatory cytokines IL-6 and IL-1β. Conversely, CAPE upregulated the expression of neuroprotective factor hemeoxygenase (HO)-1 and neurotrophic factor erythropoietin (EPO) in microglia [155].

Propolis also protected neurons from oxidative damage caused by H_2_O_2_ [156]. Propolis prevented the loss of cell viability by H_2_O_2_ and, more importantly, reversed the inhibition of brain-derived neurotrophic factor (BDNF)-induced Arc expression caused by Aβ and IL-1*β*. Propolis also induced the expression of BDNF mRNA and Arc mRNA [156]. BDNF and Arc are crucial in maintaining synaptic efficacy and plasticity and cognitive function [157,158].

The neuroprotective effect of propolis was demonstrated in various animal studies. Naware et al. (2017) showed that propolis reversed the negative effect of β-amyloid-induced cognitive impairment in mice. They found that propolis attenuated the damaging effect of β-amyloid by increasing the activity of SOD and CAT, and GSH level, while reducing maloaldehyde level [159]. The neuroprotective effect of propolis by restoring the antioxidant system was also shown by Bazmandegan et al. (2017) [160]. Furthermore, the neuroprotective effect of propolis was also demonstrated in its ability to increase the level of catecholamines, norepinephrine, dopamine, and 5-hydroxy tryptamine, and reduce the activity of acetylcholinesterase [159].

In addition, individual propolis-derived compounds exert neuroprotective properties by activating different pathways. Cadmium (Cd)-induced neurotoxicity and neurodegeneration was attenuated by CAPE in mice [161]. CAPE increased the survival rate of CdCl_2_-damaged neurons and reduced CdCl_2_-induced apoptosis. CAPE reduced the accumulation of Aβ and phosphorylated tau (p-tau) protein, and inhibited the expression of inflammatory markers such as TLR4, IL-6, IL1-β, and TNF-α [161]. In addition, chrysin exerts a neuroprotective mechanism by inhibiting NF-κB activation through a separate mechanism: upregulation of the A20 enzyme [154]. The A20 enzyme is a ubiquitin-editing enzyme that is crucial in maintaining the homeostasis of the central nervous system by regulating NF-κB signaling and its pro-inflammatory cytokines in microglia, neurons, and astrocytes [162].

Zhu et al. (2018) demonstrated that propolis was beneficial in preventing cognitive decline to mild cognitive impairment in elderly people that lived at a high altitude in a 2-year trial period [163]. The placebo group showed a decline in the Mini-Mental State Examination (MMSE) over the trial period, 26.17 to 23.87, whereas the propolis group showed an upward trend from 26.00 to 28.19. The MMSE scores correlated with the serum pro-inflammatory cytokines of the participants. Over the trial period, the serum level of IL-1β, IL-6, and TNF-α increased in the placebo arm and decreased in the propolis arm. The placebo arm showed an increase in serum IL-1β, IL-6, and TNF-α by ~182%, 155%, and 62%, respectively, whereas the propolis arm showed a decrease in serum IL-1β, IL-6, and TNF-α by ~58%, 43%, and 50%, respectively, over the 24-month trial period [163]. This confirms the importance of the anti-inflammatory action of propolis in its neuroprotective capacity. Table 1 summarizes the potential therapeutic uses of propolis in MetS and its chronic diseases.

## 7. Discussion

MetS and its associated chronic diseases appear to have common denominators, namely, chronic inflammation and increased oxidative stress. Inflammatory pathways such as NF-κB, Jun N-terminal kinase (JNK), and inflammasomes have been shown to play a significant role in the development of MetS pathophysiology [164,165,166,167,168]. Propolis has been demonstrated to modulate the inflammatory signaling pathways. CAPE, a propolis-derived compound, completely inhibited the TNF activation of the NF-κB signaling pathway. CAPE also prevents the nuclear translocation of the p65 subunit of NF-κB [169]. In addition, CAPE was shown to inhibit inducible nitric oxide synthase (iNOS), an important inflammatory enzyme, by directly modulating the NF-κB signaling pathway [170]. CAPE inhibits the transcription and synthesis of IL-2, an important NF-κB-promoting cytokine [171]. CAPE is also effective in ameliorating the calcification in human aortic valve interstitial cells by inhibiting the AKT/NF-κB/NLRP3 inflammasome pathway [172].

In addition, propolis downregulates the expression of pro-inflammatory cytokines, such as TNF-α, IL-1β, IL-4, IL-6, and IL-18 [173,174,175]. Propolis-derived compounds such as artepillin C, chrysin, 3′,4′-dihydroxy-4-methoxydalbergione, 4-methoxydalbergion, and cearoin are efficacious in inhibiting NF-κB and JNK signaling pathways [176,177,178]. Furthermore, propolis ameliorates adiponectin downregulation in adipocytes by inhibiting the TNF-α-induced JNK signaling pathway [179]. The correlation of lowered adiponectin and increased pro-inflammatory cytokine level was observed in MetS patients [180]. Propolis supplementation was efficacious in reducing various pro-inflammatory cytokines in chronic inflammation and MetS, namely, C-reactive protein (CRP), TNF-α, and IL-6 in human clinical trials [181,182].

Metabolic syndrome increases the production of AGEs and reactive oxygen species (ROS) [183,184,185,186]. The combination of these pathophysiological changes causes insulin resistance, metabolic dysfunction, oxidative stress, and mitochondrial dysfunction, leading to cell death [187,188,189,190]. AGEs are a group of modified proteins, lipids, or nucleic acids, of which their amino groups react with the carbonyl group of a reducing sugar, and subsequently become glycated and/or oxidized [191]. The interaction of AGEs with their receptor (RAGE) induces ROS production and activates the undesirable inflammation signaling pathways, such as NF-κB [192].

Propolis and its derived compounds have anti-AGE properties. Pinocembrin, a propolis-derived flavanone, inhibits the expression of the receptors for advanced glycation end products (RAGE) [193]. Propolis also directly reduces the in vitro formation of AGEs with its derived compounds, such as pinobanksin-3-acetate, 2-acetyl-1,3-dicoumaroylglycerol, pinobanksin, prenyl caffeate, and pinobanksin-5-methyl ether, being the most effective [194]. In addition, propolis in the form of nanoparticles was shown to inhibit the glycation and fructation of hemoglobin by glucose and fructose, respectively [195]. Propolis appears to have two main pathways in inhibiting AGE formation: by trapping the dicarbonyl intermediates (pinobanksin derivatives) and by activating antioxidant mechanisms (caffeic acid derivatives) [196].

Propolis exerts antioxidant properties by scavenging ROS and activating cellular antioxidant systems. Propolis extracts from various regions have been demonstrated to have radical-scavenging properties. The activity correlates with the polyphenol and flavonoid content [89,197,198,199,200]. Moreover, propolis and its phenolic constituents promote endogenous cellular antioxidant systems through various mechanisms. Propolis increases the expression of antioxidant enzymes, such as CAT, GPx, GR, GST, and SOD, and endogenous GSH [103,114,134,135,136]. Propolis also activates the important nuclear factor E2-related factor 2 (Nrf2) antioxidant signaling pathway [201,202].

**Figure 1:**
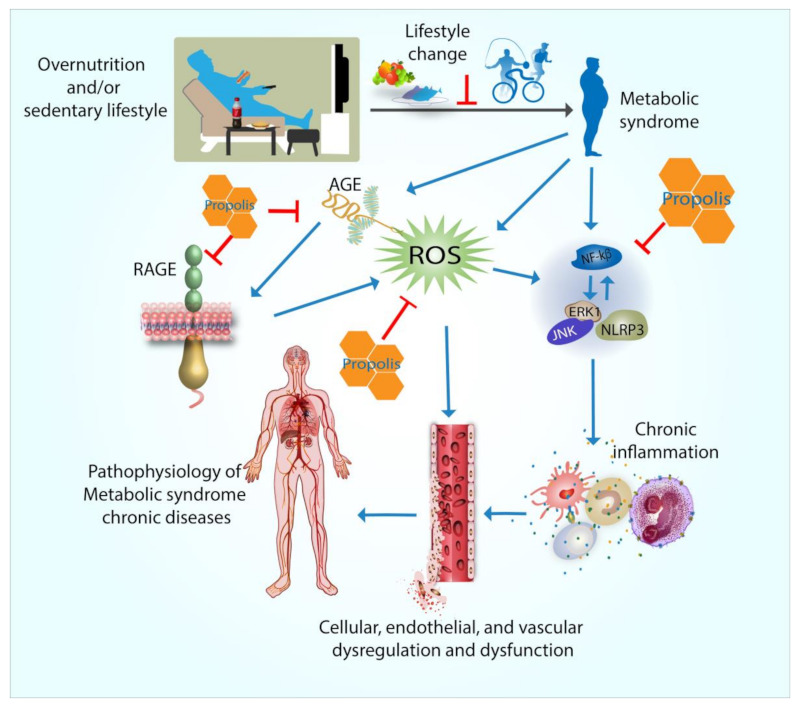
The mechanisms of action of propolis in ameliorating metabolic syndrome (MetS). MetS is induced by overnutrition and/or sedentary lifestyle. The first step of preventing MetS from developing is lifestyle modification. Once developed, MetS induces the formation of advanced glycation end products (AGEs), oxidative stress in the form of increased level of reactive of oxygen species (ROS), and chronic inflammation. AGEs bind to their receptors (RAGEs) and consequently induce further ROS production. ROS promote cellular dysfunction and dysregulation by either directly affecting the cells or inducing chronic inflammation. Chronic inflammation manifests in increased levels of pro-inflammatory cytokines through the upregulation of NF-κB, ERK, JNK, and NLRP3 inflammasome signaling pathways. Chronic and prolonged cellular, endothelial, and vascular dysfunction and dysregulation from increased AGEs, ROS, and inflammation result in the pathophysiology of MetS and its diseases. Propolis with its antioxidant and anti-inflammatory properties acts by directly inhibiting AGEs and RAGEs, ROS production, and the inflammation signaling pathways.

Figure 1 summarizes the mechanisms of action of propolis in ameliorating MetS symptoms. Sedentary lifestyle and overnutrition cause the development of MetS. One of the MetS hallmarks is the increase in blood glucose, which promotes the formation of AGEs. AGEs subsequently bind to their respective receptors (RAGEs). These interactions promote the formation of ROS. MetS also simultaneously induces the formation of ROS independent of the AGE–RAGE interactions. The increased ROS level consequently promotes the inflammatory signaling cascades through the upregulation of NF-κB, ERK, JNK, and NLRP3 inflammasome signaling pathways. The activation of these inflammatory pathways increases the level of circulating pro-inflammatory cytokines, such as C-reactive protein (CRP), TNF-α, and IL-6. The combination of increased oxidative stress and chronic inflammatory conditions causes cellular, endothelial, and vascular dysregulation and dysfunction, which manifest in the pathophysiology of MetS chronic diseases. Propolis appears to inhibit the development and manifestation of MetS by at least three mechanisms of action: the inhibition of the expression of AGE and RAGE and their interactions, the downregulation of pro-inflammatory signaling cascades, and the upregulation of the cellular antioxidant systems.

One of the main issues in propolis research and its eventual use as a therapeutic is the significant variability of its chemical composition. The chemical composition of propolis is dependent upon many variables. Several studies have shown the significant differences in chemical compositions and hence the differences in biological activities by the propolis collected by different bee species [203,204]. Geographical location also plays a significant role in the propolis chemical compositions. Comprehensive reviews by Rivera-Yanez et al. (2021) and Dezmirean et al. (2021) showed the variety of chemical compounds in propolis from around the world [205,206]. The differences in chemical composition are also evident in the propolis obtained from the same genus of plants but from different geographical locations [206].

In addition, the biological activities of propolis appear to be affected by seasonal variation. For example, Valencia et al. (2017) demonstrated that propolis obtained in different seasons from the Sonoran region (Mexico) had different chemical compositions and different antiproliferative activities against the the B-cell lymphoma cancer cell line M12.C3.F6 [207]. The antiproliferative activity (IC_50_) ranged from 11.6 µg/mL to 54.5 µg/mL, from spring to autumn, respectively [207]. Reguiera et al. (2017) also noted seasonal variation in the antimicrobial effect of Brazilian red propolis against *Escherichia coli* and *Staphylococcus aureus*, but not *Pseudomonas aeruginosa* [208]. Furthermore, the solvents used in the extract preparation play a significant role in determining the biological activities of propolis. As an example, Mishima et al. (2005) showed that 25% ethanol extract of propolis was more efficacious in terms of antihypertensive activity compared to 70% ethanol extract [81]. We propose that every study investigating the biological activities of propolis should be accompanied by chemical analysis data and geographical indications of the propolis source. The adoption of these parameters will significantly increase the reproducibility rate and the quality of propolis research going forward. A higher reproducibility rate and research quality will expedite the translation from laboratory benches to clinical uses of propolis.

## 8. Conclusions

Propolis is a sticky mixture of plant resins collected by the bees mixed with digestive enzymes of the bees, and also sometimes beeswax, that has strong antimicrobial properties that help protect the bee colony. Large numbers of in vitro, animal, and, more importantly, human clinical trials have demonstrated the safety and efficacy of propolis. The present review study illustrates the potential use of propolis in ameliorating MetS and its diseases. The therapeutic benefits of propolis in MetS appear to be associated with its potent anti-inflammatory and antioxidant properties. One of the main drawbacks of propolis as a therapeutic involves the lack of standardization. However, it can promptly be overcome by the chemical fingerprinting of its phenolics, flavonoids, terpenoids, and other bioactive compounds. The standardization (or the lack thereof) issues can also be overcome by assigning geographical indications.

## Figures and Tables

**Table 1:** Summary of various in vitro, in vivo, and human clinical trials with regard to the therapeutic uses of propolis in MetS and its associated chronic diseases.

Diseases	Types of Study	Measured Outcome	References
Cardiovascular diseases (CVDs)	in vitro and in vivo	Reduction in the triglyceride and LDL level, LDL oxidation, platelet aggregation, and expression of atherogenic growth factors and immune-mediated inflammation.	[62,63,64,65,66,67,68,69,70,71]
Atherosclerosis	human clinical trials	Increase in HDL-c concentration and antioxidant status.	[72,73,74,75,76]
Hypertension	in vivo	Reduction in hypertension, oxidative stress, inflammatory markers, and increase in antioxidant status.	[81,82,83,84,85,86,87,88,89]
Type 2 diabetes mellitus	in vivo	Improvement in blood glucose metabolism, insulin sensitivity, and antioxidant status.	[97,98,99,100,101,102,103]
	human clinical trials	Improvement in blood glucose metabolism, insulin sensitivity, antioxidant status, periodontal status, and reduction in inflammatory markers.	[74,75,104,105,106,107]
Chronic kidney disease	in vitro and in vivo	Reduction in inflammatory markers and fibrosis. Improvement in antioxidant status and renal function.	[103,114,115,116,117,118,119,120,121,122,123,124]
	human clinical trials	Reduction in proteinuria, oxidative stress, and inflammatory markers.	[125,126]
Non-alcoholic fatty liver disease	in-vitro and in-vivo	Reduction in inflammatory markers and hepatic steatosis severity. Improvement in antioxidant status.	[131,132,133,134,135,136,137,138,139,140,141,142]
	human clinical trials	Reduction in hepatic steatosis severity and inflammatory markers.	[143]
Alzheimer’s disease	in vitro and in vivo	Reduction in the Aβ and tau accumulation, and inflammatory markers. Improvement in antioxidant status.	[153,154,155,156,157,158,159,160,161,162]
	human clinical trials	Improvement in cognitive ability and reduction in inflammatory markers.	[163]
